# Respiratory muscle endurance training improves exercise performance but does not affect resting blood pressure and sleep in healthy active elderly

**DOI:** 10.1007/s00421-022-05024-z

**Published:** 2022-08-26

**Authors:** Jan Stutz, Selina Casutt, Christina M. Spengler

**Affiliations:** 1 Exercise Physiology Lab, Institute of Human Movement Sciences and Sport, ETH Zurich, Zurich, Switzerland; 2 Zurich Center for Integrative Human Physiology (ZIHP), University of Zurich, Winterthurerstrasse 190, 8057 Zurich, Switzerland

**Keywords:** Hyperpnea, Blood pressure, Exercise performance, Sleep efficiency, Hypertension, Lung function

## Abstract

**Purpose:**

Ageing is associated with increased blood pressure (BP), reduced sleep, decreased pulmonary function and exercise capacity. The main purpose of this study was to test whether respiratory muscle endurance training (RMET) improves these parameters.

**Methods:**

Twenty-four active normotensive and prehypertensive participants (age: 65.8 years) were randomized and balanced to receive either RMET (*N* = 12) or placebo (PLA, *N* = 12). RMET consisted of 30 min of volitional normocapnic hyperpnea at 60% of maximal voluntary ventilation while PLA consisted of 1 inhalation day^−1^ of a lactose powder. Both interventions were performed on 4–5 days week^−1^ for 4–5 weeks. Before and after the intervention, resting BP, pulmonary function, time to exhaustion in an incremental respiratory muscle test (incRMET), an incremental treadmill test (IT) and in a constant-load treadmill test (CLT) at 80% of peak oxygen consumption, balance, sleep at home, and body composition were assessed. Data was analyzed with 2 × 2 mixed ANOVAs.

**Results:**

Compared to PLA, there was no change in resting BP (independent of initial resting BP), pulmonary function, IT performance, sleep, body composition or balance (all *p* > 0.05). Performance significantly increased in the incRMET (+ 6.3 min) and the CLT (+ 3.2 min), resulting in significant interaction effects (*p* < 0.05).

**Conclusion:**

In the elderly population, RMET might be used to improve respiratory and whole body endurance performance either as an adjunct to physical exercise training or as a replacement thereof for people not being able to intensively exercise even if no change in BP or sleep may be expected.

## Introduction

High blood pressure (BP) is the leading cause of preventable death and disability worldwide (Forouzanfar et al. 2015). It affects more than 50% of the world adult population aged > 50 years (Mills et al. 2016). Only in a minority of cases, the aetiology of hypertension can be attributed to a specific cause while in the remaining ∼ 90%, hypertension seems to arise from a combination of genetic, environmental and behavioural factors, and has therefore been termed essential hypertension (Bolivar 2013). An important property of essential hypertension is that it is modifiable in many cases. This has important implications for normo- and prehypertensive elderly given that BP continues to increase steadily with age and since the risk of death from vascular disease has been shown to rise linearly from levels as low as 115 mmHg (systolic BP) and 75 mmHg (diastolic BP) (Lewington et al. 2002).

Lifestyle modifications, such as an increase in physical activity, have been shown to be effective strategies to lower resting BP, in both hypertensive and prehypertensive populations (Cornelissen and Smart 2013). Unfortunately, prevalence of mobility disabilities, due to acute injuries or chronic conditions, increases with age and is already high in middle age (Gardener et al. 2006). An alternative form of exercise that does not involve lower or upper limbs and that can therefore be performed independently of mobility status is respiratory muscle endurance training (RMET). When RMET is performed at near maximal intensities, respiratory muscle O_2_ consumption can account for up to 15% of whole-body maximal oxygen consumption (Aaron et al. 1992), and average heart rate (HR) during a training session can increases to ~ 60% of predicted HR_max_ in some individuals (Stutz et al. 2020). Considering that brisk walking on level ground, which can elicit similar HR responses to RMET, has been shown to lower resting BP in elderly with systolic BP > 120 mmHg (Mandini et al. 2018), one can argue that RMET intensity is sufficient to lower resting BP, but this remains to be tested. From a mechanistic point of view, RMET might positively affect BP via mechanisms intrinsic to aerobic training, via mechanisms relating to respiratory training, or a combination thereof. Specifically, since RMET involves repetitive contractions of upper body skeletal muscles resulting in an increase in HR, stroke volume (SV) and mean arterial pressure (MAP) (Rodrigues et al. 2020), we speculate that the resulting increase in shear stress lowers total peripheral resistance (TPR) and therefore MAP, a mechanism proposed to explain the beneficial effects of aerobic exercise on BP (Pescatello et al. 2004). More importantly, the large intra-arterial pressure swings generated during each inspiration and expiration and sensed by the carotid and aortic baroreceptors might lower TPR and MAP secondary to adaptations in baroreflex sensitivity (BRS). This is supported by the finding of increased BRS in a rodent model of heart failure after 6 weeks of breathing through a resistor for 30 min day^−1^ (Jaenisch et al. 2011) and by inspiratory muscle training (IMT) studies that found decreased resting BP after weeks of IMT in hypertensive and prehypertensive participants (Ferreira et al. 2013; Vranish and Bailey 2016). The first and primary aim of this study was therefore to investigate the effects of RMET on resting BP in healthy elderly. We hypothesised that four weeks of training would reduce resting BP and that a decrease of TPR secondary to an increase in BRS would be, at least in part, responsible for this effect.

In addition, exercise performance was assessed since aging is also associated with structural and functional changes of the lungs, including increased residual volume and functional residual capacity, loss of elastic recoil, increased work of breathing and decreased respiratory muscle strength (Janssens et al. 1999). During exercise, these changes lead to greater respiratory work and dyspnoea for a given intensity in elderly compared to younger subjects (Roman et al. 2016). Elderly also experience a decrease in leg muscle mass, strength, and endurance. While changes in leg muscle properties can be improved with whole body exercise training even at old age, lung function cannot be improved meaning that ventilation may become more limiting for exercise performance in older active individuals (Roman et al. 2016). Further, given that ventilation is higher when walking/running uphill compared to exercise on level ground (Pokan et al. 1995), the ventilatory system might become even more limiting under these conditions. Since RMET has been shown to improve respiratory muscle strength and endurance, exercise performance and ratings of perceived breathlessness and respiratory exertion during exercise (HajGhanbari et al. 2013; Illi et al. 2012), the secondary aim of this study was to evaluate the effects of RMET on uphill exercise performance in healthy active elderly. We hypothesised that RMET would increase endurance performance and decrease the sensation of breathlessness and respiratory exertion during exercise.

Furthermore, sleep was investigated as the prevalence of subjective sleep complaints (Foley et al. 1995) and snoring (Stoohs et al. 1998) increases with age, both affecting more than 50% of the elderly over 65 years of age. Besides, sleep complaints among elderly are often associated with increased respiratory symptoms, physical disabilities, depressive symptoms and poorer self-perceived health (Foley et al. 1995). Given that (a) whole body exercise training improves sleep quality in healthy participants irrespective of exercise modality and intensity (Kredlow et al. 2015) and (b) interventions targeting respiratory muscles such as RMET and didgeridoo playing have been shown to reduce snoring (Furrer-Boschung 1997; Puhan et al. 2006), we hypothesised that 4–5 weeks of RMET would improve sleep quality in a healthy elderly cohort likely including both snorers and non-snorers.

## Methods

### Participants

Twenty-four healthy active elderly subjects participated in this study. Baseline characteristics assessed at the first laboratory visit are shown in Table 1. Subjects were active (≥ 150 min of moderate or ≥ 75 min of vigorous aerobic activity per week), non-smoking, not taking any medication, and refrained from caffeinated and alcoholic beverages on test days before testing, did not exercise for 24 h prior to testing and refrained from intense exercise 48 h prior to testing, and slept for at least 7 h the two nights before test days. This study was approved by the cantonal ethics committee Zurich (project ID 2018-01318) and registered on clinicaltrials.gov (NCT03730935). All participants were informed about the aim of the study and gave their written informed consent prior to any data collection. Power analysis was performed assuming a medium effect size *f* for systolic BP of 0.25 for repeated measures within-between subjects interaction, power of 0.8 and an alpha level of 0.05. We chose a medium effect size of *f* = 0.25 although previous studies resulted in larger effect sizes (DeLucia et al. 2018; Ferreira et al. 2013; Vranish and Bailey 2016). The resulting sample size was 24 individuals.

**Table 1:** Participant characteristics (*N* = 12 per group)

	RMET		PLA	*P*
Gender (m/f)	8/4		7/5	0.673
Age (y)	65.0 ± 4.6		66.6 ± 5.2	0.424
Height (m)	1.71 ± 0.10		1.71 ± 0.10	0.966
Weight (kg)	73.2 ± 12.8		73.9 ± 13.9	0.897
BMI (kg m^−2^)	24.9 ± 2.9		25.2 ± 3.1	0.811
Rel. FM (%)	26.1 ± 7.5		29.3 ± 8.2	0.361
BMD (g cm^−2^)	1.11 ± 0.14		1.14 ± 0.18	0.709
Systolic BP (mmHg)	123.0 ± 11.4		122.3 ± 7.9	0.854
Diastolic BP (mmHg)	76.6 ± 7.7		76.3 ± 4.3	0.923
Handgrip (kg)	40.9 ± 8.4		43.2 ± 11.3	0.574
*V̇*O_2peak_ (mL min^−1^ kg^−1^)	33.4 ± 7.2		29.8 ± 5.7	0.186
*V̇*O_2peak_ (%predicted)	114 ± 22		111 ± 25	0.801
VC (%predicted)	112 ± 11		121 ± 15	0.098
FVC (%predicted)	107 ± 9		115 ± 15	0.136
FEV_1_ (%predicted)	112 ± 12		114 ± 11	0.666
MVV (%predicted)	126 ± 24		123 ± 17	0.706

Data are shown as mean ± SD

*RMET* respiratory muscle endurance training, *PLA* placebo, *BMI* body mass index, *FM, Rel. RM* relative fat mass, *BMD* bone mineral density, *BP* blood pressure, *MAP* mean arterial pressure, *V̇O*_*2peak*_ peak oxygen consumption, *VC* vital capacity, *FVC* forced vital capacity, *FEV*_*1*_ forced expiratory volume in the 1st second, *MVV* maximal voluntary ventilation

*P* *p* values from independent *t* tests or a chi-square test (gender)

### Study protocol

A quasi-randomized parallel group design was used to test the effects of RMET compared to placebo. Participant visited the laboratory on five different occasions. On the 1st and 2nd visits, baseline measurements were performed (pre), on the 3rd visit (~ 2 weeks into the intervention), a supervised training took place, and during the 4th and 5th visits, tests of the 1st and 2nd visits were repeated (post). Pre-post visits were scheduled at the same time of day (± 1 h) to avoid any influence of circadian rhythm. For every participant, the same investigators were present at each laboratory visit.

*1st visit*. Resting BP, height, weight, subjective sleep quality and daytime sleepiness were assessed first. Then, airway resistance, lung function, respiratory muscle strength, handgrip strength and maximum exercise performance in an incremental treadmill test (IT) were determined. Afterwards, participants were familiarized with the constant load test (CLT) and the incremental respiratory muscle endurance test (incRMET). Last, participants were allocated to either the respiratory muscle endurance training (RMET) group or the placebo (PLA) group by randomly assigning every odd-numbered participant to one of the two groups with the subsequent participant being allocated to the alternative group. Monitoring devices for sleep assessment between test days and a logbook to record respiratory muscle training, placebo inhalations and whole-body exercise during the intervention phase were given to participants at the end of the test day.

*2nd visit.* At least 72 h after the 1st visit, participants returned to the laboratory. First, anthropometric data, body composition, resting BP, hemodynamics, autonomic balance, BRS and pulse wave velocity (PWV) were assessed at rest. Afterwards, participants performed the CLT followed by a 30-min break after which they performed the incRMET. Last, participants performed the first RMET session or placebo inhalation under supervision of an investigator.

*3rd visit*. After about 2 weeks of training, participants of both groups returned for a supervised training or placebo inhalation to ensure proper performance.

*4th and 5th visit.* Two to four days after the last training session or placebo inhalation, participants returned for post assessments, which were the same as during the 1^st^ and 2^nd^ visits. Again, sleep was evaluated in between the 4^th^ and 5^th^ visits, which were separated by at least 72 h.

### Assessments

#### Airway resistance, lung function, respiratory muscle strength and handgrip strength

Airway resistance and reactance were measured using the MasterScreen impulse oscillometry system (Jaeger, Hoechberg, Germany) and assessed according to current guidelines (Oostveen et al. 2003; Winkler et al. 2009). Measurements were performed in triplicates and the average thereof was used for further analyses.

Lung function was assessed according to current guidelines (Miller et al. 2005) using a metabolic cart (Oxycon pro, Jaeger, Hoechberg, Germany). During maximal voluntary ventilation (MVV) manoeuvres, participants were instructed to try to keep the same frequency pre and post. The following reference values were used for lung volumes (Quanjer et al. 2012), flows (Garcia-Rio et al. 2004; Quanjer et al. 1993), and MVV (Cherniack and Raber 1972).

Respiratory muscle strength was recorded with a respiratory pressure meter (MicroRPM, Micro Medical/CareFusion, Kent, United Kingdom). Participants performed at least three maximal inspiratory and expiratory manoeuvres until (a) the two best measurements were within 5% and until (b) the best manoeuvre was not the last one. If more than 3 manoeuvres were performed, in- and expiratory manoeuvres were alternated after 3 manoeuvres. Reference values by Enright et al. (1993) were used for maximal inspiratory and expiratory muscle strength (Enright et al. 1993) while predicted SNIP was calculated using reference equations from (Uldry and Fitting 1995).

Handgrip strength was assessed using a digital hand dynamometer (Lafayette Instrument Co, Lafayette, IN, USA) and compared to a reference Swiss population (Werle et al. 2009). Participants were sitting on a chair without backrest with knee and hip angles at ~ 90° and with their forearms placed horizontally on a table with an elbow angle of ~ 90°. Participants performed three maximal contractions starting with the dominant hand and then alternating hands. The best of the six measurements was used for further analyses.

#### Cardiovascular assessments at rest

Assessment of cardiovascular variables at rest was done with subjects lying supine on a stretcher without talking and moving. After 5 min without any assessment, hemodynamics, autonomic balance and BRS were recorded continuously during 5 min, followed by 3 BP measurements. During the last 5 min, PWV was assessed.

*Blood pressure (BP)* Systolic BP, diastolic BP and mean arterial pressure (MAP) were measured as triplicates with a 30 s pause between two consecutive measurements (timed from complete deflation of the cuff to the start of the next inflation). Measurements were performed on the right arm at the height of the heart, using an oscillometric BP monitoring device (Cardiocap™/5, Datex-Ohmeda Inc, Madison, WI, USA). The mean of the second and third measurements was used for further analyses according to the National Health and Nutrition Examination Survey analytic and reporting guidelines (Egan et al. 2010). BP data of the 1st visit were used for screening, while data of the 2nd and 5th visit were used for analyses.

*Hemodynamics and autonomic balance* Stroke volume, HR, and cardiac output (CO) were recorded continuously during 5 min with a sampling frequency of 1 kHz (Powerlab and Labchart Software version 8 (ADInstruments Ltd, Oxford, UK) using a trans-thoracic electrical bioimpedance device (PhysioFlow®, Manatec Biomedical, Petit Ebersviller, France). Skin preparation, electrode placement and calibration were performed according to manufacturer instructions. SV, HR and CO were averaged over the 5 min interval. TPR was calculated from BP and CO using the following equation: TPR = MAP CO^−1^ 80. HRV was determined during the same 5 min using the following settings: RR interval between 800 and 1500 ms, complexity between 1.0 and 1.5, very low frequency (VLF) spectrum between 0 and 0.04 Hz, low frequency (LF) between 0.04 and 0.15 Hz and high frequency (HF) between 0.15 and 0.45 Hz. Upon visual inspection, ectopic beats were excluded from analyses. The following parameters were chosen for further analysis: standard deviation of the inter-beat intervals (SDRR) as a measure of overall variability, the root mean square of successive differences between normal heart beats (RMSSD) as the time-domain measure to estimate parasympathetic activity, the high frequency (HF) band as the frequency-domain measure to estimate parasympathetic activity, the low frequency (LF) band as a surrogate of baroreflex activity and the LF/HF ratio as a measure of sympatho-vagal balance (Shaffer and Ginsberg 2017). One participant in the RMET group was excluded from LF/HF ratio analysis due to an extreme outlier (> 3—IQR above the 3rd quartile). Inclusion of the outlier in the analysis does not change the level of significance.

*Baroreflex sensitivity (BRS)* Baroreflex sensitivity was assessed using continuous BP monitoring via a plethysmographic finger cuff (Nexfin, Edwards Lifesciences, Amsterdam, Netherlands). Inter-beat intervals (IBI) and corresponding BP values were analysed using CardioSeries Software version 2.4. An up-sequence was defined as a series (≥ 3) of increase in systolic BP (≥ 1 mmHg) followed by a lengthening of the IBI (≥ 6 ms) of the subsequent heartbeat. A down-sequence was defined as a series (≥ 3) of decrease in systolic BP (≥ 1 mmHg) followed by shortening of the IBI (≤ 6 ms) (Parati et al. 1988). All up-sequences and down-sequences within the recorded 5-min period were averaged and denominated BRS + (average of all up-sequences) and BRS − (average of all down-sequences). Four participants in the RMET group were excluded from BRS + analysis (two extreme outliers, i.e. > 3—IQR above the 3rd quartile, and two participants without BP ramps). One participant in the PLA group and four participants in the RMET group were excluded from BRS- analyses (three extreme outliers, i.e. > 3—IQR above the 3rd quartile, and 2 participants without BP ramps). Inclusion of the outliers in the analyses does not change the level of significance.

*Pulse wave velocity (PWV)* Carotid-femoral PWV (PWV_CF_) was measured using a non-invasive device (Complior, Alam Medical, Vincennes, France). Measurements were performed as triplicates. If the three measurements were within 0.5 m s^−1^ and signal quality above 90%, the values were averaged for further analysis. PWV-measurements were not possible in seven participants (4 in the RMET group and 3 in the PLA group) due to inability to detect proper carotid or femoral pulse wave forms.

#### Exercise performance and respiratory muscle endurance performance

Exercise performance was assessed on a motorized treadmill (HP-Cosmos Pulsar, h/p/cosmos sports & medical GmbH, Traunstein, Germany).

*Incremental test (IT)* Maximal aerobic capacity was assessed using the Cornell modification of the Bruce protocol (Okin et al. 1986). For 2 min before the start of the protocol, participants were standing quietly on the treadmill equipped with a 12-lead electrocardiogram (MS-12 blue, Schiller AG, Switzerland), a face mask connected to a metabolic cart (Oxycon pro, Jaeger, Höchberg, Germany), and a sensor placed on their forehead for assessment of peripheral oxygen saturation (S_p_O_2_) and HR (Nellcor™, Covidien, Minneapolis, USA). Female participants started with 0% grade and a speed of 2.7 km h^−1^. After 2 and 4 min, inclination increased to 5 and 10%, respectively, with no change in speed. Thereafter, incline and speed increased by 1% and 0.7 km/h every 2 min until volitional exhaustion. Male participants started with a 10% grade and a speed of 2.7 km h^−1^. The ECG was recorded continuously and ventilation, gas exchange, S_p_O_2_ and HR were recorded breath by breath. Breathlessness (BR; the sensation of “not getting enough air”), respiratory exertion (RE; “work/effort that is required for breathing”) and leg exertion (LE; “work/effort that is required for walking/running”) were assessed with a visual analogue scale (VAS) before the start of the IT, 30 s before the end of each 2-min stage, and immediately after the end of the IT. The scale consisted of a 10 cm line, labelled with “None” on the left end point and with “Maximal” at the right end point. A drop (20 µl) of arterialized venous blood was collected from an earlobe before the start, immediately after the end, as well as 2 and 5 min after the end of the IT. Blood lactate concentration was determined enzymatically (BIOSEN C line Sport®, EKFdiagnostic, Barleben, Germany). The switch from walking to running was kept constant between the 1st and 4th visit. Verbal encouragement during the IT was standardized in order to minimize bias (Andreacci et al. 2002).

*Constant load test (CLT)* The CLT protocol consisted of walking (RMET, *N* = 8; PLA, *N* = 9) or running (RMET, *N* = 4; PLA, *N* = 3) to volitional exhaustion on the treadmill at a constant intensity (grade and speed) corresponding to the intensity at 80% of *V̇*O_2peak_ achieved during the IT at visit 1. Participants did not shift between running and walking within and between CLTs. The test was preceded by 2-min resting baseline measurements while participants were standing quietly on the treadmill and a 2-min warm up phase at 50% of *V̇*O_2peak_. During and after the CLT, the same measurements were performed as during the IT except that subjective ratings were collected every 3 min.

*Analysis of exercise peformance* Peak exercise responses for both IT and CLT were defined as (a) the highest (ventilation, gas exchange, HR) or lowest (S_p_O_2,_ P_ET_CO_2_) 15 s moving average within the last minute of the test, (b) subjective ratings immediately after the end of the test (BR, RE and LE), and (c) maximum increase in blood lactate concentration compared to baseline. The ventilatory anaerobic threshold (VAT) was estimated visually with the V-slope method and expressed as percentage of *V̇*O_2peak_ achieved during the respective IT. *V̇*O_2peak_ comparison to predicted values was done using reference equations from the Fitness Registry and the Importance of Exercise National Database (FRIEND Registry) (Myers et al. 2017). Predicted maximal HR was calculated using the formula 208 − 0.7 × age [y]. Average values were calculated from minute 0 of the test (without warm up phase) until the end of the shorter test between pre and post (defined from now on as isotime). One participant had to be excluded from CLT analyses because the treadmill protocol stopped after 30 min at visit 2. Nonetheless, the participant performed the same 30 min protocol on day 5 for submaximal comparisons. Also, in the IT analyses, one participant in the PLA group was excluded from lactate analyses due to missing baseline value, two participants in the PLA group were excluded from HR, peak BR, RE and LE analyses due to missing data and one participant in the PLA group was excluded from RER, V̇CO_2_ and P_ET_CO_2_ analyses due to an erroneous CO_2_ calibration before the post test. Inclusion of the latter participant does, however, not change the level of significance. In the CLT analysis, one participant in each PLA and RMET group was excluded from HR analyses and one participant in the PLA group was excluded from peak BR, RE and LE analyses due to missing data.

*Incremental respiratory muscle endurance test (incRMET)* Respiratory muscle endurance was tested using the incRMET described in detail elsewhere (Vincent et al. 2016). Briefly, participants performed normocapnic hyperpnea using partial rebreathing (SpiroTiger®, idiag AG, Fehraltorf, Switzerland). The test started at a minute ventilation of 24% MVV_predicted_ (35 × FEV_1_) with constant tidal volume (bag size: 40% of vital capacity [VC], breathing frequency adjusted accordingly), and continued with ventilation increasing every 3 min (via increase in frequency) by an increment corresponding 8% MVV_predicted_ until volitional exhaustion or until the third warning by the investigator. A warning was defined as the inability to maintain tidal volume of 50% VC (with a 5% margin of error) for 5 consecutive breaths. Ventilation, gas exchange, S_p_O_2_ and HR were recorded continuously breath by breath. BR and RE were assessed before the start of the incRMET, 30 s before the end of every stage and immediately after the end of the incRMET with a VAS. Blood lactate was collected from the earlobe before the start of the test and immediately after and 2 and 5 min after the end of the incRMET. Peak and average until isotime responses were calculated in the same way as for the treadmill tests. One participant in the PLA group was excluded from analyses because of coughing and consequent inability to perform the test at visit 5. In addition, two participants in each PLA and RMET group were excluded from HR analyses, one participant in the RMET group was excluded from S_p_O_2_ analyses, and one participant in each PLA and RMET group was excluded from peak BR and RE analyses due to missing data.

#### Sleep

Sleep variables at night were assessed using wrist-worn actigraphy (Actiwatch Score, Cambridge Neurotechnology, Cambridge, UK) and pulse oximetry (WristOx2, Nonin medical, Plymouth Minnesota, USA). Furthermore, tiredness at bedtime, quality of sleep and recovery after waking up were assessed using a 10-cm VAS. During sleep assessment, participants were requested to go to bed and to get up at their usual times and to behave (i.e. eating, drinking, physical activity) as they normally do. Time in bed (TIB), sleep efficiency (SE), sleep onset latency (SOL) and fragmentation index (FI) from actigraphy and basal S_p_O_2_ (peripheral oxygen saturation during non-event times, with event defined as a drop in S_p_O_2_ by at least 4% for a minimum duration of 10 s), adjusted Index (number of events per hour) and average HR during the night from pulse oximetry were used for further analyses. Whole night sleep data was averaged over three nights, except when only two nights were recorded (due to low battery or participants forgetting to wear the device), in which case an average of two nights was used. In addition, subjective sleep quality was assessed at visits 1 and 4 using the Pittsburgh Sleep Quality Index (PSQI) and daytime sleepiness with the Epworth Sleepiness Scale (ESS). One participant in the PLA group was excluded from oximetry analyses due to missing data.

#### Anthropometrics, body composition, postural stability and physical activity

Height and weight were measured at visit 1, 2 and 5 using a stadiometer and a digital scale (Tanita BC-545 N, Tanita Europe BV, Amsterdam, The Netherlands). Data from visit 1 was only used for inclusion assessment while measurements from visits 2 and 5 were used for further analyses. Segmental fat and lean body mass (relative to total body mass) was assessed using a calibrated lunar iDXA densitometer (GE Healthcare, Madison, WI, USA). Data was analysed according to the instructions of the manufacturer. Neck and chest circumferences were measured with a non-elastic measuring tape. Neck circumference was measured just below the laryngeal prominence, with subjects standing upright and facing forward. Chest circumference was measured at the height of the xiphoid notch and with subjects raising their hands up over their heads. Measurements were performed in duplicates and the average thereof was used for further analyses. Two participants in the PLA group were excluded from bone mineral density analyses due to hip replacements.

*Postural stability* Postural stability was assessed with a balance board (MFT S3-Check, TST Trend Sport Trading GmbH, Großhöflein, Austria) before and 2 min after both the CLT and the incRMET. Participants were instructed to stand barefoot on the board and to try to keep it as horizontal as possible, minimizing left–right movements. They were allowed to move their arms and to slightly bend their knees during the trials. Measurements were performed in duplicates, each lasting 30 s with a 30 s break in between. For each trial, a stability score from 1 (very good) to 9 (very poor) was automatically calculated by the MFT software. The better trial was used for further analyses. All subjects familiarized with the device for 5 min at the first laboratory visit. Postural stability was assessed in order to evaluate the effects of fatiguing respiratory muscles on balance performance on an unstable support surface and whether RMET improves these variables. To do so, the difference in stability score before and after both the incRMET and the CLT was calculated. Negative values thus indicate lower stability after the incRMET or CLT compared to before the respective test. In addition, postural control during the incRMET was assessed using centre of pressure recordings on a force place (Kistler 3D Force plate, Kistler Group, Winterthur, Switzerland). Participants were instructed that postural control measurements would be performed for 30 s in the middle of each stage and to try to stand as stable as possible during this interval. Calculation of the 100% ellipse was done automatically by the corresponding software (MARS, Kistler Group, Winterthur, Switzerland). The average area of the ellipses until isotime was used for further analyses. One participant was excluded from balance board analyses because this participant did not perform the incRMET at visit 5. Three participants in the PLA group and one in the RMET group were excluded from postural control analyses due to missing data.

*Physical activity* The short version of the international physical activity questionnaire (IPAQ) was used to check inclusion criteria at visit 1 and to compare physical activity levels during the last 4 weeks before visits 1 and 4. In addition, a HR monitor (Polar, Kempele, Finland) and a training diary were provided to the participants during the study period to record physical activity between the first and last laboratory visits.

### Interventions

*Respiratory muscle endurance training (RMET).* Respiratory muscle endurance training consisted of 30 min of normocapnic hyperpnoea (SpiroTiger®). Training was performed on 4–5 days per week (2 days of training followed by 1 day of rest) for 4–5 weeks. Target ventilation was initially set at 60% of the individual maximal voluntary ventilation (MVV). Tidal volume (50–60% of vital capacity) and breathing frequency (calculated as 60% MVV divided by tidal volume) were held constant. Participants were instructed to increase training intensity during the training period: if they felt that during the last 5 min of a training session they (a) would be able to comfortably sustain the breathing frequency, they had to increase frequency by 2 breaths min^−1^ and start the next training with that new frequency, (b) would be able to barely sustain the frequency, they had to leave the frequency as it was and start the next training with a frequency of 1 breaths min^−1^ higher than the previous training and (c) would not be able to sustain the frequency, they had to decrease frequency by 2 breaths min^−1^ and start the next training with the frequency of the previous training. Participants were asked to keep track of RMET training intensity, perceived breathlessness and respiratory exertion (both on a scale from 0 to 10) in their training log. Training adherence was checked on a regular basis by comparing data in the participants’ personal training log with training requirements.

*Placebo (PLA)* Placebo training consisted of one inhalation of 5.5 mg lactose powder using a mock asthma inhaler (HandiHaler®, Boehringer Ingelheim, Ingelheim, Germany). This inhalation was performed 4–5 times a week for 4–5 weeks. Participants were instructed to inhale the powder according to inhaler instructions and to then perform one full inspiration to total lung capacity using custom-made, low-resistance tubing, which elicits minimal resistance to breathing. They were told that the content of the inhaler helped dilating the airways during inspiration and—when chronically applied—it would improve respiratory muscle and whole body exercise performance by reducing the work of breathing. Participants were asked to keep track of inhalations in their training log.

### Data analysis and statistics

*Participant characteristics* Distribution of the data was first checked for normality using the Kolmogorov–Smirnov (K–S) and Shapiro–Wilk (S–W) tests and homoscedasticity using Levene’s test. Since normality and homoscedasticity were not violated (*p* > 0.05), independent *t* tests (and a chi-squared test for gender) were used to compare baseline characteristics of the two groups.

*Pre-post analyses* Cardiovascular variables at rest, peak and average exercise responses, sleep, respiratory parameters, anthropometrics, body composition and postural stability were analysed using 2 × 2 mixed ANOVAs. The within subject factor was *time* with 2 levels (pre, post) and the between subject factor was *group* with 2 levels (RMET, PLA). Post-hoc simple effect analyses were further used to evaluate the effect of *Time* for each group separately (Bonferroni adjusted paired t-tests) and the effect of *group* for each timepoint separately (Bonferroni-adjusted independent *t* tests). Linear regression analyses were performed to investigate the association between time to exhaustion after the CLT and respiratory exertion.

Significance was set at *p* = 0.05 for all analyses. ANOVAs, simple effect analyses and linear regression analyses were performed using IBM SPSS Software version 25 (IBM Corp., Armonk, NY, USA).

## Results

### Interventions

Participants in the RMET group performed 21.2 ± 1.7 (mean ± SD) training sessions with a duration of 28.6 ± 1.2 min, resulting in a total training time of 10.1 ± 0.8 h. Average minute ventilation increased from 56%MVV during the first training session to 68%MVV during the 20st training session. Average HR during the RMET session performed in the laboratory was 91 ± 11 min^−1^, corresponding to 56 ± 7% of the maximal HR achieved during the IT at visit 1. Participants in the placebo group did 19.6 ± 0.5 inhalations (range 19–20).

### Respiratory parameters and handgrip

Lung function, impedance, and respiratory muscle strength and endurance are shown in Table 2. In brief, there were no significant interaction effects for neither lung function, impedance, or strength (all *p* > 0.099). Time to exhaustion during the incRMET increased significantly in the RMET group, resulting in a significant interaction effect, *F*(1, 21) = 20.38, *p* = 0.000. The same holds true for peak breathing frequency, *F*(1, 21) = 6.64, *p* = 0.018, peak minute ventilation, *F*(1, 21) = 15.32, *p* = 0.001, and peak HR, *F*(1, 17) = 13.19, *p* = 0.002 (data not shown). Average sensations of BR and RE during the incRMET decreased significantly in the RMET group, although the interaction effect did not reach statistical significance (*p* > 0.054, data not shown). There was no change in handgrip strength in either group (data not shown).

**Table 2:** Lung function, respiratory muscle strength and endurance, and airway impedance before and after respiratory muscle endurance training (RMET) and placebo inhalations (PLA)

	RMET		PLA		Mixed ANOVA		
	Pre	Post	Pre	Post	*p*. Inter	Group	Time
VC (L)	4.33 ± 0.88	4.35 ± 0.88	4.52 ± 1.03	4.52 ± 0.98	0.726	n.s	n.s
FVC (L)	4.13 ± 0.81	4.18 ± 1.00	4.30 ± 1.04	4.39 ± 1.03	0.640	n.s	n.s
FEV_1_ (L)	3.34 ± 0.70	3.36 ± 0.77	3.29 ± 0.77	3.24 ± 0.66	0.312	n.s	n.s
FEV1/FVC (%)	83.2 ± 3.8	82.6 ± 4.9	80.7 ± 6.3	78.5 ± 6.6	0.342	n.s	n.s
PEF (L s^−1^)	8.4 ± 2.3	8.8 ± 2.8	8.0 ± 1.6	8.0 ± 1.5	0.218	n.s	n.s
PIF (L s^−1^)	7.1 ± 1.7	7.5 ± 1.8	6.6 ± 1.7	6.7 ± 1.6	0.346	n.s	n.s
MVV (L min^−1^)	138 ± 40	147 ± 47	130 ± 33	140 ± 37 *	0.985	*p* < 0.001	n.s
SNIP (cmH_2_O)	81 ± 27	79 ± 27	78 ± 38	73 ± 38	0.737	n.s	n.s
MIP (cmH_2_O)	101 ± 25	96 ± 21	99 ± 35	101 ± 35	0.099	n.s	n.s
MEP (cmH_2_O)	169 ± 46	158 ± 40	152 ± 44	151 ± 33	0.195	n.s	n.s
TTE_incRMET_ (min)	21.4 ± 4.0	27.7 ± 2.7*** ^$$$^	18.8 ± 4.7	20.3 ± 3.2	0.000	*p* < 0.001	*p* < 0.01
R_5_ (kPa s L^−1^)	0.25 ± 0.07	0.26 ± 0.08	0.25 ± 0.08	0.25 ± 0.07	0.241	n.s	n.s
X_5_ (kPa s L^−1^)	− 0.08 ± 0.03	− 0.08 ± 0.03	− 0.08 ± 0.04	− 0.08 ± 0.04	0.582	n.s	n.s

Data are shown as mean ± SD

*VC* vital capacity, *FVC* forced vital capacity, *FEV*_*1*_ forced expiratory volume in the 1st second, *PEF* peak expiratory flow, *PIF* peak inspiratory flow, *MVV* maximal voluntary ventilation, *SNIP* sniff nasal inspiratory pressure, *MIP* maximal inspiratory pressure, *MEP* maximal expiratory pressure, *TTE*_*incRMET*_ time to exhaustion respiratory muscle endurance test, *R* resistance, *X* reactance, *p. Inter.* *p* value of interaction effect from mixed ANOVA

^*^p < 0.05, ***p < 0.001, within group comparison, Bonferroni adjusted paired *t* test

^$$$^*p* < 0.001, between group comparison, Bonferroni adjusted paired *t* test

### Resting cardiovascular parameters (primary outcome)

**Fig. 1:**
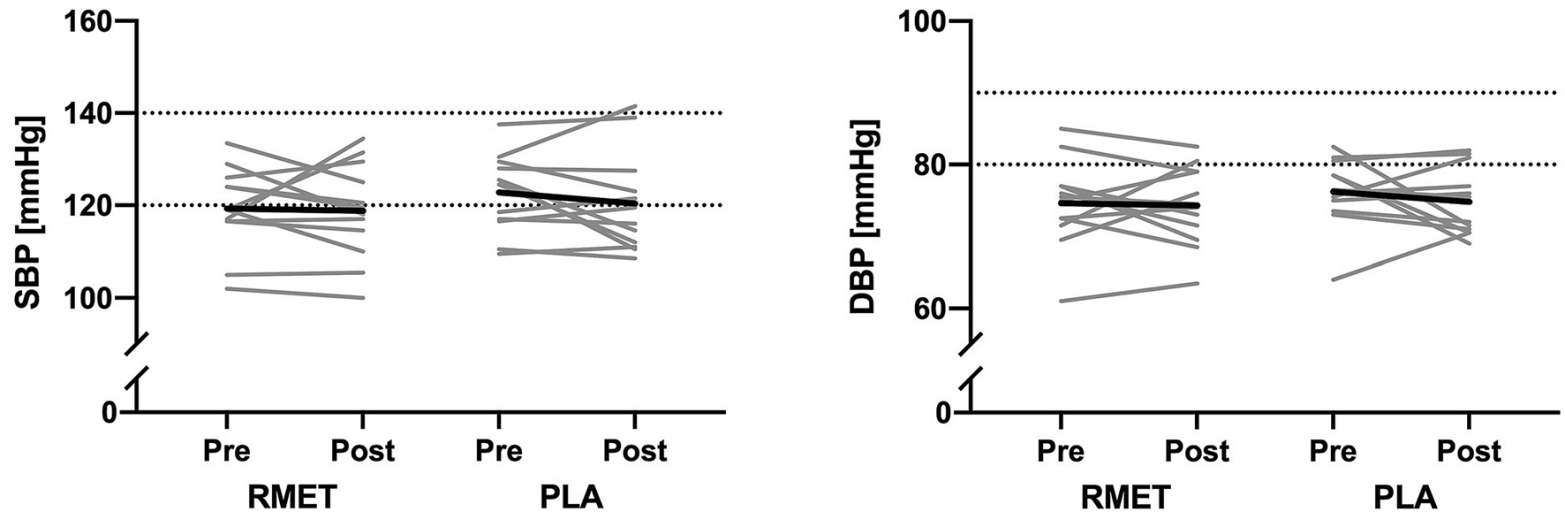
Systolic and diastolic blood pressure before and after respiratory muscle endurance training (RMET) and placebo inhalations (PLA). *Grey lines* show individual data and *black lines* give group means. *SBP* systolic blood pressure, *DBP* diastolic blood pressure

BP and cardiovascular parameters are shown in Fig. 1 and Table 3. In brief, there were no significant interaction effects for neither BP, hemodynamics, TPR, HRV, autonomic balance, PWV, nor BRS (all *p* > 0.306).

**Table 3:** Cardiovascular variables at rest before and after respiratory muscle endurance training (RMET) and placebo inhalations (PLA)

	RMET		PLA		Mixed ANOVA		
	Pre	Post	Pre	Post	*p*. Inter	Time	Group
Systolic BP (mmHg)	119.3 ± 9.1	118.8 ± 10.4	122.8 ± 8.4	120.4 ± 10.9	0.572	n.s	n.s
Diastolic BP (mmHg)	74.6 ± 6.1	74.3 ± 5.5	76.2 ± 4.9	74.8 ± 4.7	0.606	n.s	n.s
MAP (mmHg)	89.4 ± 6.3	89.2 ± 7.7	92.4 ± 6.1	91.0 ± 7.1	0.617	n.s	n.s
HR (min^−1^)	60.0 ± 7.8	58.4 ± 6.4	59.6 ± 6.0	58.7 ± 6.6	0.769	n.s	n.s
SV (mL)	88 ± 24	89 ± 22	81 ± 14	87 ± 11	0.441	n.s	n.s
CO (L min^−1^)	5.2 ± 1.2	5.2 ± 1.2	4.8 ± 0.8	5.1 ± 0.8	0.462	n.s	n.s
TPR (dyn s cm^−5^)	1431 ± 285	1428 ± 246	1582 ± 322	1448 ± 207	0.306	n.s	n.s
SDRR (ms)	48.2 ± 26.1	60.1 ± 37.8	39.1 ± 28.7	42.3 ± 19.2	0.430	n.s	n.s
RMSSD (ms)	41.6 ± 33.6	57.9 ± 54.8	33.4 ± 35.5	37.9 ± 23.2	0.377	n.s	n.s
LF (n.u.)	47.3 ± 21.3	45.5 ± 25.6	56.2 ± 24.1	56.1 ± 19.5	0.880	n.s	n.s
HF (n.u.)	50.0 ± 17.2	50.4 ± 20.8	41.9 ± 21.3	43.1 ± 16.7	0.923	n.s	n.s
LF/HF ratio	1.3 ± 1.3	1.0 ± 0.9	2.0 ± 1.5	1.8 ± 1.6	0.886	n.s	n.s
PWV_CF_ (m s^−1^)	9.1 ± 2.3	8.2 ± 3.7	8.7 ± 1.5	8.3 ± 1.6	0.591	n.s	n.s
BRS + (ms mmHg^−1^)	8.9 ± 2.9	12.0 ± 3.5 *	8.4 ± 3.0	10.3 ± 5.2	0.518	*p* < 0.01	n.s
BRS – (ms mmHg^−1^)	10.0 ± 3.3	12.6 ± 5.1 *	9.7 ± 4.5	11.2 ± 3.4	0.598	n.s	n.s

Data are shown as mean ± SD

*BP* blood pressure, *MAP* mean arterial pressure, *HR* heart rate, *SV* stroke volume, *CO* cardiac output, *TPR* total peripheral resistance, *SDRR* standard deviation of inter-beat-interval, *RMSSD* root mean square of successive differences between normal heartbeats, *LF* low frequency band, *HF* high frequency band, *PWV*_*CF*_ carotid-femoral pulse wave velocity, *BRS* baroreflex sensitivity, *p. Inter.* *p* value of interaction effect from mixed ANOVA

^*^p < 0.05, within group comparison, Bonferroni adjusted paired *t* test

### Exercise performance (secondary outcome)

**Fig. 2:**
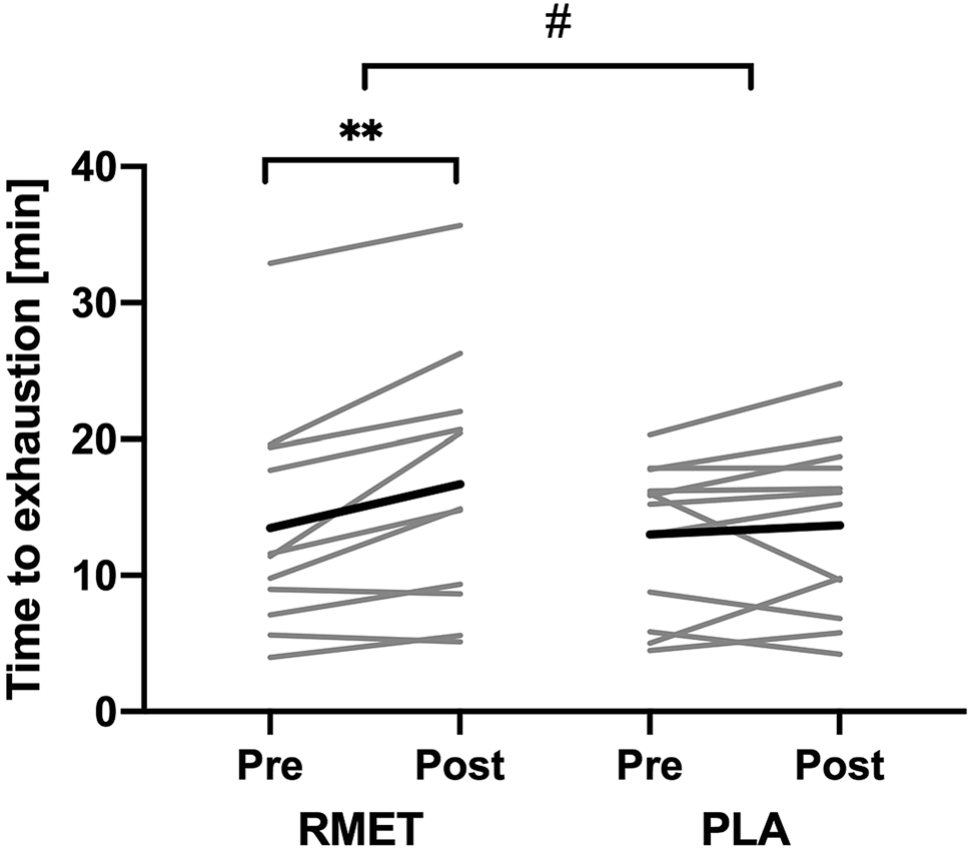
Time to exhaustion during the constant load treadmill test (CLT) before and after respiratory muscle endurance training (RMET) and placebo inhalations (PLA). *Grey lines* show individual data and *black lines* give group the mean*.* **p < 0.01, within group comparison, paired *t* test; ^**#**^*p* < 0.05, mixed ANOVA, interaction effect

**Fig. 3:**
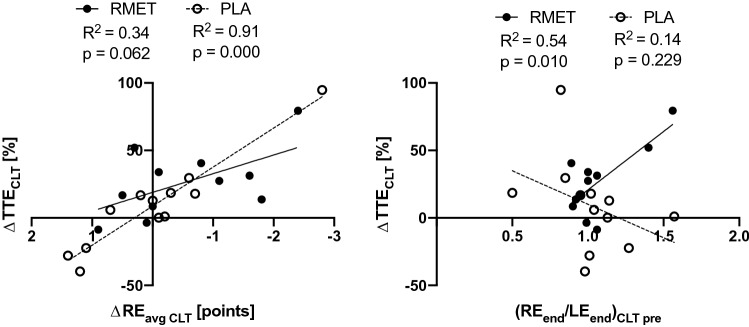
Relationship between constant load test (CLT) performance and respiratory exertion. *Left* relative change (post vs. pre) in time to exhaustion (TTE) vs. change in respiratory effort (RE) averaged until isotime. *Right* relative change (post vs. pre) in TTE vs ratio RE and leg exertion (LE) at the end of the CLT performed on the 2nd visit (pre). *RMET* respiratory muscle endurance training, *PLA* placebo

There was no difference between RMET and PLA in peak exercise responses during the IT (Table 4). TTE, *V̇*O_2peak_ and peak tidal volume increased in both groups, resulting in a significant main effect of time (all *p* < 0.050), with no difference between groups. TTE during the CLT slightly increased in the PLA group (*p* = 0.882) and increased significantly in the RMET group (*p* = 0.008), resulting in a significant interaction effect *F*(1, 21) = 4.33, *p* = 0.050 (Fig. 2). There was no other difference in peak or average exercise response between RMET and PLA during the CLT (Table 5). The increase in TTE was negatively correlated with a decrease in RE_avg_ in the placebo group (*r* = − 0.95, *p* = 0.000) and (although only a trend) in the RMET group (*r* = − 0.58, *p* = 0.062). The increase in TTE also significantly correlated with RE_end_/LE_end_ after CLT_pre_ in the RMET group (*r* = 0.73, *p* = 0.010) but not in the PLA group (*r* = − 0.38, *p* = 0.229, Fig. 3). The ratio RE_end_/LE_end_ after CLT_pre_ was used to quantify whether participants felt that respiratory exertion was more limiting to performance (ratio > 1) or leg exertion (ratio < 1).

**Table 4:** Peak exercise responses during the incremental test before and after respiratory muscle endurance training (RMET) and placebo inhalations (PLA)

	RMET		PLA		Mixed ANOVA		
	Pre	Post	Pre	Post	*p*. Inter	Time	Group
TTE (min)	12.1 ± 2.6	12.4 ± 2.5*	11.1 ± 2.2	11.4 ± 2.2	0.597	*p* < 0.01	n.s
HR (min^−1^)	158 ± 11	157 ± 12	159 ± 10	157 ± 12	0.550	n.s	n.s
*V̇*O_2_ (ml min^−1^ kg^−1^)	33.4 ± 7.2	35.3 ± 7.8	29.8 ± 5.7	31.1 ± 6.6	0.691	p < 0.05	n.s
VAT (%*V̇*O_2peak_)	56.8 ± 8.4	55.2 ± 10.3	59.6 ± 10.2	59.0 ± 8.6	0.735	n.s	n.s
RER	1.18 ± 0.07	1.19 ± 0.08	1.19 ± 0.08	1.21 ± 0.05	0.742	n.s	n.s
∆Lac (mmol L^−1^)	4.9 ± 1.6	5.0 ± 1.5	5.6 ± 1.5	5.1 ± 1.3	0.059	n.s	n.s

Data are shown as mean ± SD

*TTE* time to exhaustion, *HR* heart rate, *V̇O*_*2*_ oxygen consumption, *VAT* ventilatory anaerobic threshold, *RER* respiratory exchange ratio, ∆*Lac* maximum increase in blood lactate from baseline, *p. Inter.* *p* value of interaction effect from mixed ANOVA

^*^p < 0.05, within group comparison, Bonferroni adjusted paired *t* test

**Table 5:** Peak and average exercise responses during the constant load test (CLT) before and after respiratory muscle endurance training (RMET) and placebo inhalations (PLA)

	RMET		PLA		Mixed ANOVA		
	Pre	Post	Pre	Post	*p*. Inter	Time	Group
Peak exercise responses							
TTE (min)	13.5 ± 8.4	16.7 ± 9.5**	13.0 ± 5.5	13.7 ± 6.3	0.050	*p* < 0.01	n.s
HR (min^−1^)	160 ± 12	162 ± 13	159 ± 11	157 ± 11	0.114	n.s	n.s
*V̇*O_2_ (mL min^−1^ kg^−1^)	33.5 ± 7.6	35.1 ± 8.1	31.6 ± 4.4	32.0 ± 6.9	0.436	n.s	n.s
RER	1.08 ± 0.1	1.04 ± 0.09	1.08 ± 0.08	1.07 ± 0.11	0.341	n.s	n.s
∆Lac (mmol L^−1^)	5.1 ± 2.2	4.9 ± 2.3	5.4 ± 2.4	5.2 ± 2.6	0.934	n.s	n.s
Averages until isotime							
HR (min^−1^)	147 ± 11	145 ± 13	147 ± 11	143 ± 9	0.474	*p* < 0.05	n.s
*V̇*_E_ (L min^−1^)	80 ± 25	79 ± 27	79 ± 24	76 ± 25	0.474	n.s	n.s
*V*_T_ (L)	2.6 ± 0.7	2.6 ± 0.8	2.4 ± 0.7	2.3 ± 0.7	0.659	n.s	n.s
f_R_ (min^−1^)	32 ± 9	31 ± 8	33 ± 4	33 ± 5	0.762	n.s	n.s
*V̇*O_2_ (mL min^−1^ kg^−1^)	30.7 ± 6.6	31.7 ± 7.5	28.7 ± 4.2	28.7 ± 5.8	0.435	n.s	n.s
RER	1.02 ± 0.06	1.00 ± 0.06	1.04 ± 0.05	1.02 ± 0.07	0.702	n.s	n.s
BR (points)	4.2 ± 2	3.6 ± 2.3	4.6 ± 1.8	4.5 ± 2.2	0.375	n.s	n.s
RE (points)	6.0 ± 1.7	5.3 ± 1.9	5.2 ± 2.2	5.1 ± 2.2	0.189	n.s	n.s
LE (points)	5.6 ± 1.7	5.7 ± 1.6	5.0 ± 2.2	5.2 ± 2.3	0.884	n.s	n.s

Data are shown as mean ± SD

*TTE* time to exhaustion, *HR* heart rate, *V̇O*_*2*_ oxygen consumption, *RER* respiratory exchange ratio, ∆*Lac* maximum increase in blood lactate from baseline, *V̇*_*E*_ minute ventilation, *V*_*T*_ tidal volume, *f*_*R*_ breathing frequency, *BR* breathlessness, *RE* respiratory exertion, *LE* leg exertion, *p. Inter.* *P* value of interaction effect from mixed ANOVA

^**^p < 0.01, within group comparison, Bonferroni adjusted paired *t* test

### Sleep (secondary outcome)

Sleep variables are shown in Table 6. There was no significant change in sleep after RMET (all *p* > 0.144). SE and PSQI decreased in both groups, resulting in a significant main effect of time (both *p* < 0.050). There was an interaction effect for subjective feeling of recovery after waking up, *F*(1, 22) = 5.26, *p* = 0.032, due to a non-significant decrease in the PLA group and a slight increase in the RMET group.

**Table 6:** Sleep before and after respiratory muscle endurance training (RMET) and placebo inhalations (PLA)

	RMET		PLA		Mixed ANOVA		
	Pre	Post	Pre	Post	*p*. Inter	Time	Group
ESS (0–24)	5.3 ± 3.7	5.7 ± 3.1	5.9 ± 3.1	5.2 ± 3.2	0.246	n.s	n.s
PSQI (0–21)	4.5 ± 2.8	3.3 ± 1.4	4.8 ± 3.6	4.3 ± 3.0	0.338	*p* < 0.05	n.s
TIB (h)	7.8 ± 0.3	7.8 ± 0.6	7.8 ± 0.8	7.5 ± 0.7	0.246	n.s	n.s
SE (%)	89.0 ± 2.4	85.3 ± 9.1	87.1 ± 6.9	82.8 ± 10.6 *	0.836	*p* < 0.05	n.s
SOL (min)	7.6 ± 5.4	11.6 ± 15.3	9.6 ± 8.5	17.4 ± 19.6	0.574	n.s	n.s
Fragmentation index	27 ± 10	33 ± 16	30 ± 16	31 ± 20	0.378	n.s	n.s
Tiredness (points)	5.5 ± 1.6	5.7 ± 2.1	6.0 ± 1.2	6.4 ± 1.4	0.658	n.s	n.s
Sleep quality (points)	5.5 ± 1.6	5.7 ± 2.1	6.8 ± 1.6	6.0 ± 2.4	0.071	n.s	n.s
Recovery (points)	7.3 ± 1.6	7.5 ± 1.3	7.5 ± 1.5	6.7 ± 2.3	0.032	n.s	n.s
Adj. index (events h^−1^)	6.0 ± 5.3	5.9 ± 4.6	5.3 ± 4.4	5.1 ± 4.6	0.971	n.s	n.s
HR_avg_ (min^−1^)	59.5 ± 6.4	59.7 ± 7.2	60.7 ± 4.8	59.4 ± 6.0	0.317	n.s	n.s

Data are shown as mean ± SD

*ESS* Epworth sleepiness scale, *PSQI* Pittsburgh sleep quality index, *TIB* time in bed, *SE* sleep efficiency, *SOL* sleep onset latency, *Adj. index* desaturation index, *HR*_*avg*_ average heart rate during the night, *p. Inter.* *p* value of interaction effect from mixed ANOVA

^*^p < 0.05, within group comparison, Bonferroni adjusted paired *t* test

### Anthropometrics, body composition, postural stability and physical activity

There was no significant interaction effect for height, weight, relative and absolute fat mass, relative and absolute fat-free mass, bone mineral density, neck and chest circumferences and physical activity levels (all *p* > 0.114, data not shown). Postural stability was not compromised by either CLT or incRMET, nor was this modified by RMET or PLA. Average ellipse area of the centre of pressure during the incRMET decreased in both groups, resulting in a significant main effect of time (*p* < 0.050, data not shown) without an interaction effect.

## Discussion

To the best of our knowledge, this is the first placebo-controlled study to investigate the effects of RMET on BP, exercise performance and sleep in healthy active elderly. After 4–5 weeks of RMET, neither BP nor sleep changed, while constant-load uphill exercise performance significantly improved.

### Blood pressure

Four to five weeks of RMET had no effect on resting systolic and diastolic BP (− 0.5 and − 0.3 mmHg). Although, in prehypertensive participants of the RMET group (*N* = 5), systolic BP decreased by 4.8 mmHg and diastolic BP by 3.1 mmHg, but the same or larger changes were seen in prehypertensive of the PLA group (*N* = 7) where resting systolic and diastolic BP decreased by 4.7 and 4.2 mmHg, respectively. These results emphasize again the importance of a placebo group, especially in BP studies, where robust placebo effects are observed (Wilhelm et al. 2016).

Why did RMET have no effect on BP considering that IMT has often been shown to lower systolic and diastolic BP in hypertensive (Ferreira et al. 2013), prehypertensive (Vranish and Bailey 2016) and even normotensive (DeLucia et al. 2018; Vranish and Bailey 2015) participants by 5–10 mmHg? Part of this discrepancy may result from different types of analyses, e.g. Ferreira and colleagues report a significant decrease in 24-h ambulatory systolic BP after IMT (− 7.9 mmHg) but also a non-significant decrease in the placebo group (approx. 3–4 mmHg), without providing any between-group comparison. Also, not all of the IMT studies found lower resting BP values after a training intervention (Mills et al. 2015). A recent meta-analysis found, in fact, that overall, BP was not altered with IMT (Cipriano et al. 2019), although this analysis included only six studies. Nonetheless, the following comparison with IMT studies that found a decrease in BP might provide an explanation as to why we did not see a change in BP in the current study.

*Age of participants* The age-associated increase in systolic BP has been suggested to be associated with structural alterations of the cardiovascular system that do not seem to be reversible by habitual physical activity (Jakovljevic 2018), thus potentially limiting positive adaptations to training. However, studies showed that BP is decreased to a similar extent after whole body exercise in younger (< 50 years) and older (> 50 years) subjects (Cornelissen and Smart 2013). Also, several studies showed a decrease in resting BP in cohorts of older participants after IMT (Ferreira et al. 2013; Vranish and Bailey 2016), showing that BP can indeed be improved in older age.

*Intrathoracic pressure* While during IMT large negative intrathoracic pressures of about − 45 to − 60 mmHg are observed (Vranish and Bailey 2015), during RMET, pressure swings do not seem to exceed 20 mmHg (Schaer et al. 2019). More importantly, these large intrathoracic pressure swings seem to be necessary for BP adaptations, as Vranish and Bailey elegantly showed (Vranish and Bailey 2015). Although we were aware of the modest intrathoracic pressures that are observed during RMET, we speculated that BP adaptations might be greater because of the larger acute increase in systolic BP seen during RMET compared to IMT. This seemed reasonable because the proposed link between acute increases in intrathoracic pressure and long-term BP reduction is an enhanced sensitivity of baroreceptors (Ferreira et al. 2013; Vranish and Bailey 2015, 2016; Craighead et al. 2019) that are stimulated by acute changes in BP. The finding of an increase in vagal modulation and a decrease in sympathetic outflow after 8 weeks of IMT in hypertensive participants (Ferreira et al. 2013) provides indirect support of BRS adaptations after IMT. Indeed, BRS in a rodent model of heart failure has been shown to be improved after 6 weeks of breathing through a resistor for 30 min day^−1^ and 5 days week^−1^ (Jaenisch et al. 2011). However, this finding has not yet been clearly confirmed in humans as BRS did not change after 6 weeks of IMT in healthy active adults (DeLucia et al. 2018), while in our cohort after 4–5 weeks of RMET it increased within the RMET group but was not different from PLA, pointing towards random variation and/or placebo effects. It appears that other/additional factors are necessary for BRS adaptation, such as an increase in compliance of arteries in which the baroreceptors are located, which in turn can increase afferent responsiveness (Hagg et al. 2004; Laterza et al. 2007). Given that elevated shear stress is an important stimulus for vascular adaptations (Green et al. 2017), the hemodynamic stimulus imposed by RMET in this study might therefore have been too low to result in measurable changes in arterial function and BRS.

### Exercise performance

Participants in the RMET group improved CLT performance by 24%, resulting in a significant interaction effect. This improvement occurred in the absence of changes in maximal aerobic capacity assessed with an IT. Although also IT performance increased in the RMET group, there was no difference when compared to PLA. The finding of an improved CLT performance without changes in IT performance is consistent with previous literature (Illi et al. 2012). The absence of RMET effects on IT performance can likely be explained by the fact that exercise duration above an intensity of 85% *V̇*O_2max_ (a threshold for respiratory muscle fatigue development proposed by Johnson et al. 1993) is too short in the IT to elicit significant respiratory muscle fatigue (Illi et al. 2012). On the other hand, other respiratory muscle training (RMT) studies fail to show improvements in constant-work exercise tests that exceed placebo and/or learning effects (Sonetti et al. 2001). As explained below and also elucidated by Sonetti and colleagues, these differential effects might be dependent on the tested population and the choice of exercise tests.

The 24%-increase in CLT performance is larger than the average improvement seen with previous RMET studies, ranging from 0 to 25% (Fairbarn et al. 1991; Holm et al. 2004; McMahon et al. 2002; Morgan et al. 1987; Stuessi et al. 2001; Verges et al. 2008, 2007), although direct comparison is somewhat limited by the fact that these studies all used cycling as exercise modality, whereas we used a treadmill protocol. The relatively large increase in TTE could possibly be attributed to the tested population (i.e. active elderly whose exercise performance might be limited by the ventilatory system) and/or the protocol employed (i.e. uphill treadmill walking/running, known to impose a greater stress on the ventilatory system compared to level ground walking/running). Data of Aznar-Lain and colleagues support this suggestion as these authors also found large increases in uphill CLT performance (TTE + 36%) after 8 weeks of IMT in healthy and moderately active elderly (Aznar-Lain et al. 2007). However, more research is needed to investigate the relationship between uphill exercise performance, ventilatory system limitation and RMT. Potential adaptations that might explain the increase in exercise performance seen in this study are discussed below.

*Cardiovascular adaptations* Whole body exercise training improves exercise performance by increasing blood volume, submaximal and maximal SV, maximal CO, blood flow to active muscles, by widening the arteriovenous oxygen difference and by decreasing resting and submaximal heart rate (Kenney et al. 2015). However, the increase in endurance performance after RMET is likely to be attributable to other factors as we found no change in resting, submaximal or maximal HR, SV, and CO. This is further supported by Markov and colleagues who found that changes in submaximal HR, SV and substrate utilization were only present after whole body aerobic training but not after RMET (Markov et al. 2001).

*Metaboreflex* Attenuation of the respiratory metaboreflex has been proposed to explain the ergogenic effects of RMT (Witt et al. 2007). Briefly, the metaboreflex theory states that fatiguing contractions of respiratory muscles during high intensity exercise increases sympathetic outflow, thereby limiting leg blood flow and exercise performance (Dempsey et al. 2006). Increased tolerance to respiratory muscle fatigue would therefore lead to attenuation of the reflex and result in lower sympathetic outflow, increased leg blood flow and endurance performance. Having not measured respiratory muscle fatigue, muscle sympathetic nerve activity (MSNA) and leg blood flow, we cannot confirm nor disprove that changes in the respiratory metaboreflex were responsible for the increase in exercise performance. Worth of mention here is that quadriceps fatigue, but not diaphragm fatigue, has been shown to occur in healthy sedentary elderly after exhaustive cycling exercise (Mador et al. 2000), speaking against the metaboreflex theory in our cohort. However, the authors concede that this might be different in active elderly whose performance is more likely to be limited by the ventilatory system.

*Respiratory muscle performance* The 31%-increase in respiratory muscle endurance after RMET was not correlated with the increase in TTE during the CLT, speaking against the hypothesis that increased respiratory endurance is solely responsible for the increase in whole body exercise performance. Possibly, perception of breathing also played a role. The unaltered respiratory muscle strength after RMET was expected given the specificity of training adaptations in general and in respiratory muscles, i.e. RMET typically results in improvements of respiratory muscle endurance, while IMT results in increased respiratory muscle strength (HajGhanbari et al. 2013).

*Sensation of respiratory exertion* In both RMET and PLA groups, the change in average RE and the change in TTE during the CLT were negatively correlated (although only a trend in the former). This indicates that a reduced perception of RE was associated with an increased TTE. Since only in the RMET group, a tendency towards a decrease in average RE (− 0.7 points) was observed during the CLT, this subtle change could potentially have added to the improved endurance performance. This decrease in RE was apparent at all timepoints and with similar magnitude during the CLT, although never reaching statistical significance (paired t-tests, data not shown). Further, participants who rated their RE to be higher than their LE after the baseline CLT were also the ones who improved most after RMET (see Fig. 3). However, more research is needed to confirm the assumption that subjects that feel more limited by their respiratory system are the ones that profit the most from RMET, as the correlation mainly results from 2 participants.

In conclusion, the exact mechanisms to why RMET improved exercise performance remain unknown, but factors involving perceptions of RE, attenuation of respiratory muscle fatigue and the respiratory muscle metaboreflex seem to be involved.

### Sleep

Respiratory muscle endurance training did not result in better sleep in healthy elderly. The interaction effect observed for the perception of recovery after sleep can be explained by the decrease seen in the PLA group and by the small increase in the RMET group. Surprisingly, there was a main effect of time also for SE, being lower after the RMET period. This is due to four participants in the PLA group and three participants in the RMET group who showed large decreases in SE after the training period for reasons possibly unrelated to the intervention. However, we did not exclude these participants from analyses because we cannot exclude the possibility that this is indeed due to the RMET or PLA intervention. The improvement seen in PSQI score after both RMET and PLA points towards a placebo effect or random variation (given the large number of analyses).

A possible explanation for the lack of effect of RMET on sleep is that participants had normal ESS (Johns 1991) and PSQI scores (Smyth 2008), higher than normal TST and SE values and lower than normal SOL values (Ohayon et al. 2004) already at baseline, thus potentially being subjected to ceiling effects. Furthermore, only one participant in each group reported loud snoring at least once a week (i.e. prevalence of 8%), which is surprisingly low for an older cohort, possibly related to the regular exercise of these subjects. Also, DeLucia and co-workers found no improvement in sleep after IMT in healthy participants with normal sleep (DeLucia et al. 2018), but sleep was improved in patients with obstructive sleep apnoea (OSA) (Vranish and Bailey 2016). However, after physical exercise, healthy volunteers with normal sleep were shown to have improved sleep according to a meta-analysis (Kredlow et al. 2015) which suggests that improvements in sleep might be expected also after RMT.

Worth of mention is that the desaturation index (DI) was slightly above normal (i.e. DI > 5 (Chung et al. 2012) in subjects of the present study. However, a sub-analysis of participants with a DI > 5 (RMET, *N* = 6; PLA, *N* = 5) also does not result in a significant interaction effect (*p* = 0.759, data not shown), suggesting no benefits of RMET in participants with a slightly elevated DI. This finding is supported by previous studies that also found unaltered apnoea/hypopnea index (AHI) in OSA patients after 5 weeks of RMET (Herkenrath et al. 2018) or 6 weeks of IMT (Vranish and Bailey 2016). Interestingly, didgeridoo playing for 4 months has been shown to improve AHI and ESS in OSA patients (Puhan et al. 2006), but this form of training is different from the classic RMET or IMT protocols given that it involves circular breathing, lip vibrations and manipulations of the tongue, throat and the diaphragm (Eley 2013). A comprehensive analysis on the potential effects of RMT on OSA was, however, out of the scope of this investigation.

### Postural stability

RMET did not influence body composition or indexes of postural stability. We had hypothesised that RMET might improve postural stability in instances when subjects’ respiratory muscles are fatigued, e.g. after exercise or volitional hyperpnea. This was based on the observations that (a) contractions of the diaphragm can contribute to the control of the trunk and thus postural control (Gandevia et al. 2002), (b) acute loading of the inspiratory muscles leads to suboptimal postural control (Janssens et al. 2013; Hodges et al. 2001), (c) fatigued inspiratory muscles increase postural sway on an unstable support surface in healthy subjects (Janssens et al. 2010) and (d) RMET increases fatigue resistance of respiratory muscles in subjects who develop > 10% of diaphragm or abdominal muscle fatigue (Verges et al. 2007). However, in the present study, balance was not compromised in any of the tasks tested. Likely, the challenge for postural muscles was not large enough in these subjects that were used to perform physical exercise as numerous other factors are involved in balance control, such as inputs from vision, proprioception and the vestibular system, integration of these signals, the corresponding motor output to the muscles, and psychological factors (Pu et al. 2015).

## Limitations

Different factors limit the generalizability and validity of the current findings. Given the characteristics of our cohort (healthy, active, systolic and diastolic BP below 140 and 90 mmHg), we cannot infer to which extent BP in a sedentary and/or hypertensive population might be affected. Also, missing data limits the interpretation of some outcomes, in particular PWV with 7 missing participants out of 24, resulting in a power of 0.60. In addition, we used non-invasive methods to determine spontaneous cardiac BRS which is less accurate than BRS assessment via injection of phenylephrine (Milic et al. 2009). Finally, conclusions about the effect of RMET on sleep are limited as sleep was assessed via home measurements and questionnaires and only three nights were used for actigraphic recording of sleep at home due to restrictions in the protocol (Aili et al. 2017).

## Conclusion

In the elderly population, RMET might be used to improve respiratory and whole body endurance performance either as an adjunct to physical exercise training or as a replacement thereof for people not being able to intensively exercise even if no change in BP or sleep may be expected.

## Abbreviations

AHI: Apnea–Hypopnea Index
BP: Blood pressure
BR: Breathlessness
BRS: Baroreflex sensitivity
CLT: Constant load test
DI: Desaturation index
ESS: Epworth sleepiness scale
FI: Fragmentation index
HR: Heart rate
HRV: Heart rate variability
IMT: Inspiratory muscle training
incRMET: Incremental respiratory muscle endurance test
IPAQ: International physical activity questionnaire
IT: Incremental test
LE: Leg exertion
MVV: Maximal voluntary ventilation
PLA: Placebo
PSQI: Pittsburgh sleep quality index
PWV: Pulse wave velocity
RE: Respiratory exertion
RMET: Respiratory muscle endurance training
RMT: Respiratory muscle training
SE: Sleep efficiency
SOL: Sleep onset latency
SV: Stroke volume
TIB: Time in bed
TTE: Time to exhaustion
VAS: Visual analogue scale
*V̇*O_2peak_: Peak oxygen consumption
